# The Association Between Electronic Device Use During Family Time and Family Well-Being: Population-Based Cross-Sectional Study

**DOI:** 10.2196/20529

**Published:** 2020-10-14

**Authors:** Sheng Zhi Zhao, Ningyuan Guo, Man Ping Wang, Daniel Yee Tak Fong, Agnes Yuen Kwan Lai, Sophia Siu-Chee Chan, Tai Hing Lam, Daniel Sai Yin Ho

**Affiliations:** 1 School of Nursing University of Hong Kong Hong Kong China (Hong Kong); 2 School of Public Health University of Hong Kong Hong Kong China (Hong Kong)

**Keywords:** eDevice, smartphone, mobile phone, well-being, family dinner, family communication, public health

## Abstract

**Background:**

Electronic devices (eDevices) may have positive or negative influences on family communication and well-being depending on how they are used.

**Objective:**

We examined eDevice use during family time and its association with the quality of family communication and well-being in Hong Kong Chinese adults.

**Methods:**

In 2017, a probability-based 2-stage random sampling landline telephone survey collected data on eDevice use in daily life and during family time (eg, family dinner) and the presence of rules banning eDevice use during family dinner. Family communication quality was rated from 0 to 10 with higher scores being favorable. Family well-being was calculated as a composite mean score of 3 items each using the same scale from 0 to 10. The associations of family communication quality and well-being with eDevice use in daily life and during family time were estimated using beta-coefficient (β) adjusting for sociodemographics. The mediating role of family communication quality in the association between eDevice use and family well-being was analyzed.

**Results:**

Of the 2064 respondents (mean age 56.4 [SD 19.2] years, 1269/2064 [61.48%] female), 1579/2059 (76.69%) used an eDevice daily for a mean of 3.6 hours (SD 0.1) and 257/686 (37.5%) used it for 30+ minutes before sleep. As much as 794/2046 (38.81%) often or sometimes used an eDevice during family time including dinner (311/2017, 15.42%); 713/2012 (35.44%) reported use of an eDevice by family members during dinner. Lower family communication quality was associated with hours of eDevice use before sleep (adjusted β=–.25; 95% CI –0.44 to –0.05), and often use (vs never use) of eDevice during family dinner by oneself (adjusted β=–.51; 95% CI –0.91 to –0.10) and family members (adjusted β=–.54; 95% CI –0.79 to –0.29). Similarly, lower family well-being was associated with eDevice use before sleep (adjusted β=–.26; 95% CI –0.42 to –0.09), and often use during family dinner by oneself (adjusted β=–.48; 95% CI –0.83 to –0.12) and family members (adjusted β=–.50; 95% CI –0.72 to –0.28). Total ban of eDevice use during family dinner was negatively associated with often use by oneself (adjusted odds ratio 0.49; 95% CI 0.29 to 0.85) and family members (adjusted odds ratio 0.41; 95% CI 0.28, 0.60) but not with family communication and well-being. Lower family communication quality substantially mediated the total effect of the association of eDevice use time before sleep (61.2%) and often use at family dinner by oneself (87.0%) and by family members (67.8%) with family well-being.

**Conclusions:**

eDevice use before sleep and during family dinner was associated with lower family well-being, and the association was substantially mediated by family communication quality. Our results suggest that interventions on smart use of eDevice may improve family communication and well-being.

## Introduction

Family well-being (also known as family life satisfaction, family welfare, and family functioning), which emphasizes the importance of health and satisfaction of family relationships, is a hallmark of individual happiness and social cohesion [1,2]. While family relationships and the sense of well-being may differ widely by family structure, good family verbal and nonverbal communication is essential regardless [3]. Family well-being in Chinese culture highlights family health, harmony, and happiness (3Hs) with family communication being a core component [4]. Socioeconomic (SES) status has been a robust predictor of family well-being, but recent studies showed that higher income only contributed to its dynamic fluctuation but not to a long-lasting increase due to hedonic adaptation and social comparison [5,6]. Family life and communication are robust predictors of family well-being and our previous trial showed that improved family interactions and communication quality enhanced family well-being [7]. However, in busy modern societies, time for family life and communication is limited.

Information communication technology has transformed interpersonal communication and lifestyle. Internet-based electronic devices (eDevices) including personal computers, tablets, and smartphones may improve well-being through increasing work efficiency and capability, easy access to information, and convenient social interactions using communication tools [8-10]. However, inappropriate use of eDevice is a significant public health issue. Addiction-like symptoms and feelings of dependence, dangerous use (eg, distracted driving), and loss of control were common symptoms of problematic eDevice use associated with various health problems [11]. Obsessive eDevice use was linked to poor sleep quality, sedentary lifestyle, physical inactivity, and obesity [12,13]. Psychological symptoms such as depression, social isolation, low self-esteem, and anxiety have also been associated with problematic eDevice use [14].

Time spent on eDevice reduces leisure and family gathering hours. There is a growing concern that eDevice may detract from, rather than complement, social interactions [15]. The act of ignoring others in favor of smartphone use at a social setting, also called phone snubbing (phubbing), has become increasingly common and is associated with poorer relationship satisfaction [15-17]. Smartphone use during social gatherings may hamper conversation and interaction [18]. As the major domain of family life in which family members express love, care, and support; share value and happiness; and resolve problems, quality family communication is vital for maintaining family well-being [19]. eDevice use, especially smartphone use, is invading traditional family functions, such as family gatherings and dinners, which threatens communication quality and hence family well-being [20,21]. Experiencing family phubbing was associated with lower levels of interpersonal relationship and perceived well-being [20]. We also found that problematic smartphone use is also associated with poorer family well-being via deprived family communication time and quality [22].

We found no reports on the relationships of eDevice use with perceived family communication quality and family well-being. eDevice use during family time (eg, family dinner) is rarely reported despite its potential link to poor well-being [15]. Some families may adopt rules banning eDevice use during family dinner, but its effect on family well-being is unclear. Hong Kong, as one of the most developed non-Western urbanized cities in China with a high internet penetration rate and a dense family living environment, provided an appropriate platform to observe and understand the eDevice use behavior in family life. We examined eDevice use in daily life, especially during family time, and its associations with family communication quality and well-being in Chinese adults in Hong Kong. The potential mediation effect of communication quality on the association between eDevice use and family well-being was analyzed. Moreover, the prevalence and effect of the rules banning eDevice use during family dinner were assessed.

## Methods

### Sampling

The Hong Kong Family and Health Information Trends Survey (FHinTs) under the FAMILY project was a probability-based cross-sectional telephone survey conducted in 2017 to monitor family health, information use, and communication among Cantonese speaking Hong Kong adults (aged 18+). All interviews were conducted by trained interviewers of the Public Opinion Programme of the University of Hong Kong. Details of the methods were reported elsewhere [10,23,24]. Briefly, adopting a 2-stage random sampling strategy, residential telephone directories that covered 76% of Hong Kong residents were used to generate population-representative telephone numbers by random. Invalid household numbers and nonresponses (after 5 calls at different times and days of the week) were excluded. The second stage was to select an eligible individual in each household with the soonest next birthday. Each interview took 25-30 minutes. Of 5449 eligible households, 4054 adults were successfully interviewed (response rate 74.40%). Of these, 2064 (50.91%) respondents were randomly selected to answer a subset of questions related to the use of computer, smartphone, or tablet (eDevice); family communication; and family well-being. The Institutional Review Board of the University of Hong Kong/Hospital Authority Hong Kong West Cluster granted ethics approval. Verbal informed consent was obtained.

### Measurements

Family included biological, marital, or cohabited relationships. eDevices included computers, smartphones, and tablets. Duration of self-reported eDevice use daily was categorized into “No use,” “1 hour or less,” “1-2 hours,” “2-3 hours,” “3-5 hours,” and “more than 5 hours.” eDevice use before sleep was categorized as “No use,” “30 minutes or less,” “31-60 minutes”, “1-2 hours,” and “more than 2 hours”. Family time referred to the time spent with family members. The frequency of eDevice use during family time was categorized as “Never,” “Seldom,” “Sometimes,” and “Often.” The same response options were used for the frequency of eDevice use during family dinner by oneself and by family members. Rules banning eDevice use during family dinner were categorized as “No ban,” “Partial ban (limited time or frequency),” and “Total ban.”

Perceived communication quality between family members was rated on a scale of 0 (very poor) to 10 (very good), which positively correlated with the Subjective Happiness Scale, Short War-wick-Edinburgh Mental Well-being Scale, and Family Functioning and Family Communication Scale (Pearson correlation coefficients [*r*] range: .37-.60, all *P*<.001) [22]. Family well-being was calculated based on the composite score of family health, harmony, and happiness (3Hs) using 3 separate questions on the same scale of 0-10, with a higher score indicating better family well-being. The Chinese version of Family Happiness was found valid and reliable among Hong Kong adults [25]. The internal consistency of family well-being in this study was excellent (Cronbach α=.89).

Sex, age, and marital status (never married, married or cohabitated, divorced or separated, and widowed) of the respondents were recorded. Education attainment was categorized as primary or below, secondary, and tertiary. Monthly household income was categorized as HK $9999 or less (US $1290 or less), HK $10,000-19,999 (US $1290-US $2580), HK $20,000-29,999 (US $2580-US $3870), HK $30,000-39,999 (US $3870-US $5161), HK $40,000 or more (US $5161 or more), and unstable [10,23].

### Data Analysis

To improve the representativeness of the sample, all descriptive data were weighted according to the sex–age distribution of the Hong Kong population in the year end of 2015 and the education attainment distribution in the 2011 census. We used chi-square tests to compare the prevalence of eDevice use by sociodemographics. We also used paired *t* test and analysis of variance to compare the average duration of daily eDevice use, family communication, and family well-being across sociodemographic status. Multivariable linear regression was conducted to calculate the coefficient of family communication quality and family well-being in relation to eDevice use during family time, adjusted for potential sociodemographic confounders including sex, age, marital status, education attainment, and income. We used the Baron and Kenny’s approach [26] to examine the mediating effect of family communication quality on the association between eDevice use and family well-being. Sobel–Goodman tests were adopted to decompose the total effects of eDevice use on family well-being. Bootstrapping with 1000 replications was used to calculate the 95% CI of the indirect effect. Logistic regression was conducted to estimate the odds ratios of sometimes or often use of eDevice during family dinner in relation to rules banning its use with the same adjustment model. A 2-sided *P*-value <.05 was considered statistically significant. All analyses were conducted using STATA 15.0 (StataCorp).

## Results

Table 1 shows that among the 2064 respondents, 1269 were female (61.48%), 1035 aged 25 to 64 years (50.15%), and 1306 married or cohabitated (63.28%). A total of 1582 respondents had secondary or higher educational attainment (76.65%) and 1032 had a monthly household income of more than HK $20,000 (US $2580; 50.00%). Respondents who were male, younger, never married, and had higher education attainment and household income used eDevice more (all *P*<.01). Family communication quality and well-being scores were higher among older adults and those who had higher household income (all *P*<.01; Multimedia Appendix 1).

**Table 1:** Sociodemographic characteristics and hours of eDevic^a^ use daily (N=2064).

Sociodemographic characteristic		Values				Prevalence of eDevice use				eDevice usage daily (N=1579 hours)			
		Crude n (%)		Weighted %^b^		Crude n (Weighted %)		*P* value		Mean (SD)		*P* value	
**Sex**								.007				.004	
	Male		795 (38.52)		45.03		634 (84.64)				3.7 (3.3)		
	Female		1269 (61.48)		54.97		945 (81.82)				3.4 (3.2)		
**Age, years**								<.001				<.001	
	18-24		213 (10.32)		9.12		211 (97.69)				5.9 (3.4)		
	25-44		297 (14.39)		35.42		290 (96.99)				5.0 (3.6)		
	45-64		738 (35.76)		36.96		655 (85.51)				3.1 (2.9)		
	≥65		816 (39.53)		18.50		423 (44.11)				1.9 (2.1)		
**Marital status**								<.001				<.001	
	Never married		445 (21.56)		29.30		423 (94.09)				5.6 (3.7)		
	Married/Cohabitated		1306 (63.28)		60.47		1010 (83.40)				2.9 (2.7)		
	Divorced/Separated		79 (3.83)		3.59		52 (69.23)				3.1 (3.2)		
	Widowed		234 (11.34)		6.64		94 (38.58)				1.9 (2.0)		
**Education attainment**								<.001				<.001	
	≤Primary		482 (23.35)		23.66		193 (50.79)				1.6 (1.6)		
	Secondary		864 (41.86)		48.09		700 (89.74)				3.0 (3.0)		
	Tertiary		718 (34.79)		28.25		686 (98.71)				4.6 (3.4)		
**Monthly household income (HK $ [US $])^c^**								<.001				<.001	
	≤9999 (≤1290)		478 (23.16)		16.04		227 (47.69)				2.3 (2.5)		
	10,000-19,999 (1290-2580)		294 (14.24)		17.00		223 (82.50)				3.1 (3.0)		
	20,000-29,999 (2580-3870)		326 (15.79)		19.72		284 (90.32)				3.6 (3.4)		
	30,000-39,999 (3870-5161)		222 (10.76)		13.01		205 (96.18)				3.8 (3.2)		
	≥40,000 (≥5161)		484 (23.45)		24.00		456 (97.22)				4.3 (3.3)		
	Unstable		260 (12.60)		10.23		184 (75.01)				3.2 (3.2)		

^a^eDevice: electronic device.

^b^Weighted by sex, age and educational attainment distribution of Hong Kong census.

^c^US $1 = HK $7.8.

Table 2 shows that 1579/2059 (76.69%) respondents used eDevice daily for 3.6 hours on average; 257/686 (37.5%) used eDevice for more than 30 minutes before sleep and 794/2046 (38.81%) sometimes or often use eDevice during family time; 311/2017 (15.42%) and 713/2012 (35.44%) reported oneself and family members sometimes or often use eDevice during family dinner, respectively. Only 376/2045 (18.39%) reported a partial or total ban of eDevice use during family dinner.

Table 3 shows that per-hour increase in using eDevice daily was not associated with family communication quality (adjusted β=–.02, 95% CI –0.04 to 0.01) and family well-being (adjusted β=–.01, 95% CI –0.03 to 0.01) after controlling for sociodemographic characteristics. Per-hour increase in eDevice use before sleep, however, was associated with lower family communication quality and family well-being (all *P* for trend <.01). Specifically, more than 2 hours of use before sleep was significantly associated with lower family communication quality (adjusted β=–1.05, 95% CI –1.86 to –0.24; *P*=.002) and family well-being (adjusted β=–.97, 95% CI –1.66 to –0.28; *P*<.001). Often use of eDevice during family dinner both by oneself and by family members was associated with lower family communication quality (adjusted β=–.54 to –0.51) and family well-being (adjusted =–.50 to –0.48). However, overall use of eDevice during family time was not associated with family communication quality (adjusted β=–.14, 95% CI –0.41 to 0.13) and family well-being (adjusted β=–.06, 95% CI –0.29 to 0.18).

**Table 2:** Frequency of eDevice^a^ use and prevalence of rules banning eDevice use during family dinner.

Content		Crude n (%)^b^	Weighted %^c^
**Time using eDevice daily (hours) (N=2059)**			
	No use	480 (23.31)	16.98
	≤1	424 (20.59)	18.74
	1-2	291 (14.13)	15.15
	2-3	237 (11.51)	11.93
	3-5	258 (12.53)	14.70
	>5	369 (17.92)	22.51
	Mean (SD) in hours among users	3.5 (3.2)	3.6 (0.1)
**Time using eDevice before sleep (N=686)**			
	No use	329 (47.96)	38.43
	≤30 minutes	100 (14.58)	16.17
	31-60 minutes	179 (26.09)	30.90
	1-2 hours	52 (7.58)	9.87
	>2 hours	26 (3.79)	4.63
	Mean (SD) in hours among users	0.94 (0.9)	0.98 (0.1)
**eDevice use during family time (N=2046)**			
	Never	867 (42.38)	33.85
	Seldom	385 (18.82)	19.48
	Sometimes	503 (24.58)	29.34
	Often	291 (14.22)	17.32
**eDevice use during family dinner by oneself (N=2017)**			
	Never	1329 (65.89)	58.26
	Seldom	377 (18.69)	21.34
	Sometimes	217 (10.76)	14.56
	Often	94 (4.66)	5.84
**eDevice use during family dinner by family members (N=2012)**			
	Never	888 (44.14)	39.46
	Seldom	411 (20.43)	22.31
	Sometimes	424 (21.07)	23.14
	Often	289 (14.36)	15.09
**Rules banning eDevice use during family dinner (N=2045)**			
	No ban	1669 (81.61)	78.34
	Partial ban^d^	201 (9.83)	11.21
	Total ban	175 (8.56)	10.46

^a^eDevice: electronic device.

^b^Sample sizes varied because of missing values on some variables.

^c^Weighted to sex, age, and educational attainment distribution of Hong Kong census.

^d^Partial ban: Limited time or frequency of use.

**Table 3:** Daily eDevice^a^ use in relation to family communication quality and family well-being.

eDevice use		Family communication quality (0-10)		Family well-being (0-10)	
		Crude β (95% CI)	Adjusted β (95% CI)^b^	Crude β (95% CI)	Adjusted β (95% CI)^b^
**Time using eDevice daily (hours)**					
	Not used	Reference	Reference	Reference	Reference
	≤1	–.14 (–0.32 to 0.04)	–.16 (–0.35 to 0.03)	–.07 (–0.23 to 0.08)	–.12 (–0.28 to 0.05)
	1-2	–.16 (–0.36 to 0.03)	–.15 (–0.38 to 0.07)	–.03 (–0.20 to 0.14)	–.07 (–0.26 to 0.13)
	2-3	–.29 (–0.51 to –0.08)^c^	–.26 (–0.50 to –0.01)^d^	–.11 (–0.29 to 0.08)	–.12 (–0.33 to 0.09)
	3-5	–.26 (–0.46 to –0.05)^d^	–.18 (–0.42 to 0.07)	–.01 (–0.20 to 0.17)	.03 (–0.19 to 0.24)
	>5	–.39 (–0.58 to –0.20)^e^	–.24 (–0.49 to 0.01)	–.26 (–0.42 to –0.09)^c^	–.16 (–0.37 to 0.06)
	Per-hour increase	–.04 (–0.06 to –0.02)^e^	–.02 (–0.04 to 0.01)	–.02 (–0.04 to –0.01)^c^	–.01 (–0.03 to 0.01)
**Time using eDevice before sleep**					
	Not used	Reference	Reference	Reference	Reference
	<30 minutes	–.26 (–0.68 to 0.16)	–.14 (–0.60 to 0.31)	–.08 (–0.44 to 0.28)	–.11 (–0.49 to 0.28)
	30-60 minutes	–.40 (–0.74 to –0.05)^d^	–.14 (–0.53 to 0.26)	–.15 (–0.45 to 0.15)	–.04 (–0.37 to 0.30)
	61-120 minutes	–.46 (–1.01 to 0.09)	–.13 (–0.72 to 0.46)	–.36 (–0.83 to 0.11)	–.19 (–0.69 to 0.31)
	>120 minutes	–1.57 (–2.33 to –0.80)^e^	–1.05 (–1.86 to –0.24)^c^	–1.32 (–1.98 to –0.67)^e^	–.97 (–1.66 to –0.28)^c^
	Per-hour increase	–0.39 (–0.57 to –0.22)^e^	–.25 (–0.44 to –0.05)^c^	–.34 (–0.49 to –0.19)^e^	–.26 (–0.42 to –0.09)^c^
**eDevice use during family time**					
	Never	Reference	Reference	Reference	Reference
	Seldom	.01 (–0.22 to 0.24)	.05 (–0.19 to 0.29)	.07 (–0.13 to 0.27)	.10 (–0.11 to 0.31)
	Sometimes	–.08 (–0.29 to 0.13)	.02 (–0.21 to 0.25)	–.06 (–0.25 to 0.12)	.02 (–0.18 to 0.22)
	Often	–.25 (–0.50 to 0.09)	–.14 (–0.41 to 0.13)	–.15 (–0.37 to 0.08)	–.06 (–0.29 to 0.18)
**eDevice use during family dinner by oneself**					
	Never	Reference	Reference	Reference	Reference
	Seldom	–.03 (–0.25 to 0.19)	.06 (–0.17 to 0.29)	–.02 (–0.21 to 0.18)	.04 (–0.15 to 0.24)
	Sometimes	–.19 (–0.47 to 0.08)	–.08 (–0.36 to 0.20)	–.12 (–0.36 to 0.12)	–.03 (–0.28 to 0.22)
	Often	–.61 (–1.01 to –0.22)^c^	–.51 (–0.91 to –0.10)^d^	–.58 (–0.93 to –0.23)^e^	–.48 (–0.83 to –0.12)^c^
**Use of eDevice during family dinner by family members**					
	Never	Reference	Reference	Reference	Reference
	Seldom	–.09 (–0.31 to 0.13)	–.01 (–0.23 to 0.21)	.00 (–0.19 to 0.19)	.06 (–0.13 to 0.26)
	Sometimes	–.29 (–0.51 to –0.08)^c^	–.24 (–0.46 to –0.02)^d^	–.29 (–0.49 to –0.10)^c^	–.25 (–0.45 to –0.06)^c^
	Often	–.55 (–0.80 to –0.30)^e^	–.54 (–0.79 to –0.29)^e^	–.51 (–0.73 to –0.28)^e^	–.50 (–0.72 to –0.28)^e^
**Rules banning eDevice use during family dinner**					
	No ban	Reference	Reference	Reference	Reference
	Partial ban^f^	–.02 (–0.30 to 0.26)	–.04 (–0.33 to 0.24)	–.11 (–0.36 to 0.14)	–.12 (–0.37 to 0.12)
	Total ban	.27 (–0.03 to 0.57)	.21 (–0.08 to 0.37)	.23 (–0.03 to 0.50)	.18 (–0.08 to 0.44)

^a^eDevice: electronic device.

^b^Adjusted for sex, age, marital status, education attainment, and family income.

^c^*P*<.01.

^d^*P*<.05.

^e^*P*<.001.

^f^Partial ban: Limited time or frequency of use.

Table 4 shows that family communication quality mediated the association of per-hour increase in eDevice use before sleep and often use of eDevice during family dinner with family well-being by meeting the Baron and Kenny’s mediation assumptions [26]. Sobel–Goodman tests showed that family communication quality mediated 61.2%, 87.0%, and 67.8% of the total effect of hourly increase in eDevice use before sleep and eDevice use by oneself and by family members during family dinner on family well-being, respectively. The total ban was not associated with family communication quality (adjusted β=.21, 95% CI –0.08 to 0.37) and family well-being (adjusted β=.18, 95% CI –0.08 to 0.44). Table 5 shows that the total ban was associated with decreased eDevice use during family dinner (adjusted odds ratio 0.49, 95% CI 0.29-0.85). Family members were also less likely to use eDevice during dinner with the total ban (adjusted odds ratio 0.41, 95% CI 0.28-0.60). Partial ban showed no effect on eDevice use or well-being.

**Table 4:** Mediation of the association between eDevice^a^ use and family by perceived family communication quality.

Communication quality		Family well-being	
		β^b^	95% CI^c^
**Hourly increase in eDevice use before sleep^d^**			
	Total effect	–.27	–0.43 to –0.10^e^
	Indirect effect (through mediator)	–.16	–0.30 to –0.02^f^
	Direct effect (without mediator)	–.10	–0.26 to 0.05
**Often use of eDevice during family dinner by oneself^ g^**			
	Total effect	–.42	–0.77 to –0.07^e^
	Indirect effect	–.28	–0.54 to –0.02^f^
	Direct effect	–.14	–0.40 to 0.12
**Often use of eDevice during family dinner by family members^h^**			
	Total effect	–.16	–0.22 to –0.09^e^
	Indirect effect	–.11	–0.16 to –0.06^e^
	Direct effect	–.05	–0.10 to –0.00^i^

^a^eDevice: electronic device.

^b^Adjusted for sex, age, marital status, education attainment and family income.

^c^Bootstrapping with 1000 replications for bias-corrected 95% confidence interval.

^d^Proportion of total effect mediated: 61.2%.

^e^*P*<.001.

^f^*P*<.01.

^g^Proportion of total effect mediated: 87.0%.

^h^Proportion of total effect mediated: 67.8%.

^i^*P*<.05.

**Table 5:** Association between eDevice^a^ use during dinner and rules banning such use.

Family rules on eDevice use		Sometimes or often use eDevice during family dinner by oneself				Sometimes or often use eDevice during family dinner by family members			
		Adjusted odds ratio (95% CI)^b^		*P* value		Adjusted odds ratio (95% CI)^b^		*P* value	
**Rules banning eDevice use during family dinner**									
	No ban		Reference				Reference		
	Partial ban^c^		1.02 (0.69 to 1.52)		.91		1.33 (0.98 to 1.81)		.07
	Total ban		0.49 (0.29 to 0.85)		.01		0.41 (0.28 to 0.60)		<.001

^a^eDevice: electronic device.

^b^Adjusted for sex, age, marital status, education attainment, and family income.

^c^Partial ban: Limited time or frequency of use.

## Discussion

### Principal Findings

This study provided the first evidence that eDevice use during family dinner was associated with lower family communication and well-being in a population-representative adult sample. Male sex, younger age, and being single were associated with longer time spent on eDevice [27]. Respondents with higher SES status may use eDevice more because of longer work-related use. In our previous study, respondents with higher SES status used eDevice more frequently for health and family information seeking and sharing [24], and communicated with family members more via phone calls, instant messaging (IM), and video calls [9,10]. Higher SES status respondents had higher access to eDevice, which may also lead to greater use [24]. As eDevices become cheaper and easier to use, such SES status difference of eDevice use may reduce. The associations between eDevice use and psychological well-being were mixed and inconclusive [28]. eDevice use has facilitated interpersonal communication through different social platforms but may intervene in face-to-face interactions at social settings. Our results were consistent with large-scale surveys and systematic reviews that higher daily use of eDevice was generally not associated with poorer well-being [29-31]. Moderate daily eDevice use may benefit well-being through more efficient social interactions and greater perceived social support [32]. Moreover, we found that excessive eDevice use before sleep was associated with lower family well-being and the association was partially mediated by a perceived lower family communication quality. Studies have reported that sleep disturbance also partially mediated the relationship between eDevice use before sleep and depression symptoms in adolescents [13].

Excessive smartphone use reduces the interaction among family members and leads to lower levels of relationship satisfaction and family cohesion and induces family conflicts [17,33,34]. However, eDevice use during dinner has become a social norm [15]. We found that often or sometimes use of eDevice was very common during family time (794/2046, 38.81%) and family dinner (311/2017, 15.42%). Family gatherings and quality communication are vital for family well-being and dinner time is the most important part of family life. Our results indicate a potential risk to fundamental social needs resulting from eDevice use during family dinner. Previous studies found that individuals who phubbed others or experienced phubbing in social settings may experience lower relationship satisfaction than those who received direct eye contact [35]. Our results showed that poor family communication quality substantially mediated the associations between eDevice use at dinner and family well-being. A perceived interruption of interaction, including verbal and nonverbal signals such as gestures and attitude, displays most common features of social exclusion, which can lead to detrimental effects on psychological social needs including belongingness, self-esteem, meaningful existence, and control [36]. In addition, negative perceived interaction quality and negative relationship satisfaction were also associated with phubbing others and may reinforce the eDevice use under the family environment [15,20]. Nevertheless, the temporal association could not be inferred in this cross-sectional study. According to the Compensatory Internet Use theory [37], people with poor family support may use eDevice to compensate for their social needs, avoid conflict, and alleviate negative emotions. The perceived lower family well-being can be supported by other psychological effects in relation to the increased use of eDevice such as feeling lonely, having poor social support, and having higher likelihood of developing depression or other mental distress [14].

Communication is the keystone of harmony and well-being of family relationship as well as the determinant of other satisfactory social connections such as friendship and partnership [3]. Our results, consistent with the literature, indicate that eDevice use at family settings may harm relationships [20]. However, we have reported that using eDevice for family communication such as family chat groups and video chat was associated with higher family communication quality and family well-being [10]. An increasing number of people are using IM for family communication. Future studies may investigate the context of eDevice use during family time. Involving and encouraging family members to use eDevice instead of excluding them from social networking could be practical for maintaining a harmonious family relationship. Seldom or sometimes use of eDevice during family time is generally not associated with lower family communication and well-being. Considering a total ban might not be feasible for every family in modern societies; instead, avoiding excessive use of eDevice would be more feasible and less likely to induce confrontations among family members. Research on community interventions to improve the awareness of the family members to use eDevice smartly for a healthy and happy family environment is warranted.

We found that 18.39% (376/2045) of Hong Kong households limited eDevice use at family dinner and a total ban was significantly associated with less use. Regulated eDevice use during family activities could provide an interactive venue to foster social awareness and improve self-regulation. A previous trial [38] has found that a family colearning program of eDevice use behavior could improve the mutual understanding of usage behavior, improve use-limiting, appreciably changed smartphone-mediated parent–child interaction, and decreased the total smartphone usage. Only 175/2045 (8.56%) of Hong Kong families had a total ban of eDevice use during dinner. Future studies might consider adopting community-based interventions to promote and facilitate family rules for greater family health and well-being. eDevice companies also recognized that being overoccupied with notifications and attached to internet-based activities could be problematic. A “do not disturb” feature has thus been developed in some smartphones (eg, iPhone; Apple) to release people from vibrations, lights, or rings for a nondisrupted meeting, conversation, dinner, and sleep. However, evidence to guide policies on appropriate eDevice exposure is limited, and mostly designed for children and adolescents. Proper family guidance is needed for a better family communication and well-being.

This study had several limitations. First, unmeasured residual confounding cannot be excluded in any observational study. Dispositional factors such as personality trait, including impulsivity and neuroticism, were predictors of problematic internet use and well-being. Major life events (eg, disability, divorce), family relationships, and social capital were also vital to subjective and family well-being but were not adjusted for [1,39,40]. Second, the cross-sectional design has restricted causal interpretation. Third, we used landline telephone directories for household sampling, and thus excluded families with only mobile phones. Mobile sample might include younger, higher educated, and high-income households [22]. However, the internet penetration in Hong Kong has reached 98% in 2017 [41], suggesting a very limited difference for eDevice access in this population. Recall bias in reporting the eDevice use cannot be eliminated. We did not separate eDevice use into computer, smartphone, or tablet use. The associations with well-being might not be significantly different between different eDevices used as they shared most commonly used features. Finally, perceived family communication quality and family well-being were assessed by questionnaires developed under Chinese social context. The generalizability of the findings with family communication and well-being warrants further study in other settings.

### Conclusion

eDevice use before sleep and during family dinner was associated with lower family well-being, and the association was substantially mediated by family communication quality. Our results suggest that interventions on smart use of eDevice may improve family communication and well-being.

## Appendix

Multimedia Appendix 1 Supplementary Table 1.

## Abbreviations

eDevice: electronic device
SES: socioeconomic
