# Supplementary material for: The Association Between Electronic Device Use During Family Time and Family Well-Being: Population-Based Cross-Sectional Study
Source: J Med Internet Res. 2020 Oct 14;22(10):e20529. doi: 10.2196/20529 (PMC7593857; doi:10.2196/20529)
Supplement: Multimedia Appendix 1 [file jmir_v22i10e20529_app1.docx]

**Supplementary table 1. Family communication quality and well-being by socio-demographic characteristics (N=2064).**

|  | **Family communication quality (0-10)** | | **Family well-being (0-10)** | |
| --- | --- | --- | --- | --- |
|  | **Mean (SD)** | ***P*-value** | **Mean (SD)** | ***P*-value** |
| **Sex** |  | .10 |  | .44 |
| Male | 6.9 (1.9) |  | 7.4 (1.6) |  |
| Female | 7.0 (1.9) |  | 7.5 (1.7) |  |
| **Age, years** |  | <.001 |  | <.001 |
| 18-24 | 6.7 (1.7) |  | 7.1 (1.6) |  |
| 25-44 | 6.8 (1.7) |  | 7.5 (1.5) |  |
| 45-64 | 6.8 (1.8) |  | 7.3 (1.7) |  |
| ≥ 65 | 7.2 (2.1) |  | 7.7 (1.7) |  |
| **Marital status** |  | <.001 |  | <.001 |
| Never married | 6.5 (1.8) |  | 7.1 (1.7) |  |
| Married / Cohabitated | 7.1 (1.8) |  | 7.6 (1.6) |  |
| Divorced / Separated | 6.4 (2.0) |  | 6.5 (2.1) |  |
| Widowed | 7.2 (2.4) |  | 7.7 (1.8) |  |
| **Education attainment** |  | .95 |  | .77 |
| ≤ Primary | 6.9 (2.3) |  | 7.5 (1.9) |  |
| Secondary | 7.0 (1.9) |  | 7.4 (1.7) |  |
| Tertiary | 6.9 (1.7) |  | 7.5 (1.5) |  |
| **Monthly household income ^b^ (HK$)** |  | .005 |  | <.001 |
| ≤ 9,999 | 6.8 (2.2) |  | 7.3 (1.9) |  |
| 10,000-19,999 | 6.7 (2.0) |  | 7.2 (1.7) |  |
| 20,000-29,999 | 6.9 (1.8) |  | 7.5 (1.6) |  |
| 30,000-39,999 | 6.8 (1.9) |  | 7.4 (1.7) |  |
| ≥ 40,000 | 7.2 (1.6) |  | 7.7 (1.4) |  |
| Unstable | 7.1 (2.0) |  | 7.7 (1.6) |  |
